# Modeling Huddling Penguins

**DOI:** 10.1371/journal.pone.0050277

**Published:** 2012-11-16

**Authors:** Aaron Waters, François Blanchette, Arnold D. Kim

**Affiliations:** Applied Mathematics, University of California Merced, Merced, California, United States of America; Institut Pluridisciplinaire Hubert Curien, France

## Abstract

We present a systematic and quantitative model of huddling penguins. In this mathematical model, each individual penguin in the huddle seeks only to reduce its own heat loss. Consequently, penguins on the boundary of the huddle that are most exposed to the wind move downwind to more sheltered locations along the boundary. In contrast, penguins in the interior of the huddle neither have the space to move nor experience a significant heat loss, and they therefore remain stationary. Through these individual movements, the entire huddle experiences a robust cumulative effect that we identify, describe, and quantify. This mathematical model requires a calculation of the wind flowing around the huddle and of the resulting temperature distribution. Both of these must be recomputed each time an individual penguin moves since the huddle shape changes. Using our simulation results, we find that the key parameters affecting the huddle dynamics are the number of penguins in the huddle, the wind strength, and the amount of uncertainty in the movement of the penguins. Moreover, we find that the lone assumption of individual penguins minimizing their own heat loss results in all penguins having approximately equal access to the warmth of the huddle.

## Introduction

Emperor penguins (*Aptenodytes forsteri*) are known to huddle to survive long periods of fast in the severe conditions of Antartcic winters. They are able to form huddles because they are not tied to a fixed nest. Huddles are discontinuous events that last for relatively short durations (on the order of a few hours) [1] corresponding to storm events [2], [3]. The number density in a huddle at a colony may be as high as 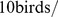
m^2^ [4], [5]. Researchers have observed directly penguins huddling at their colonies [1], [2], [5]–[7], and there is evidence indicating that emperor penguins also huddle during foraging trips [8].

Emperor penguins huddle to conserve energy, which is particularly important since they must fast for periods of 105 to 115 days [6]. Emperor penguins benefit from huddles because of the reduction of body surface area exposed to the cold and owing to the warm temperature inside the huddle [9]. The ambient temperature in the huddle is at least 20°C and may reach as high as 37.5°C [1]. Despite the fact that huddles achieve these high ambient temperatures, emperor penguins benefit most from the huddle through the reduction of cold-exposed body surfaces [10].

Measurements of the body temperature of penguins in various environments and their relation to weather conditions have led to significant insight into huddle formation. [1], [3], [7] Huddles occur more frequently at lower ambient temperatures and in higher wind speed, but the intensity of the huddle (*i.e.* the number density of the huddle) depends on lower ambient temperatures only [3]. Few theoretical models of huddling have been presented. Some modeling effort has assumed that penguins move from the windward to the leeward side of the huddle, but without providing much justification [11]. Canals and Bozinovic [12] modeled huddle formation in mice (Mus musculus) as a self-organizing event. In particular, they modeled huddling as a second-order phase transition triggered by cold temperatures.

An important feature of huddles is that each penguin has approximately equal opportunity to the warmth of the huddle. How each penguin obtains this equal access is thought to be the result of a complex phenomenon in which penguins reorganize themselves within the huddle [1]–[3]. Gilbert *et al* [1] attribute heterogeneity of the huddle shape to ensuring this equal access, but without providing details as to how equality is achieved. On the other hand, Zitterbart *et al* [13] use ideas from condensed matter physics to explain how penguins within a huddle reorganize themselves. An important set of observations regarding penguin movement within huddles reported by Le Maho [5] is

*The huddles are not motionless; movement is extremely slow, but continuous. The huddle is urged along by the wind, the rear-flank birds (those most exposed to the wind) advancing slowly along the sides of the huddle in order to be protected from the wind. Thus, birds that at first are in the center of the huddle become members of the rear flank and move, in their turn, up the sidelines.*

Further evidence showed that huddles move back and forth under the influence of the dominant winds [3].

We introduce here a systematic and quantitative mathematical model for penguin huddles. This mathematical model is aligned with the qualitative observations by Le Maho stated above. Moreover, it is consistent with the idea that penguins huddle tightly to reduce their cold-exposed body surfaces, and increase the ambient temperature. The key assumption of our mathematical model is that each individual penguin seeks to reduce its own heat loss. Thus, a penguin on the boundary of the huddle exposed to the wind will move downwind along the huddle boundary. In contrast, the penguins in the interior of the huddle neither have space to move nor experience significant heat loss, so they remain stationary. While penguins inside the huddle have been observed to make multiple small displacements [13], we consider here that these motions are small compared to the motion observed on the edge of the huddle. The accumulation of individual penguin movements along the boundary leads to coherent and robust huddle dynamics that we identify, describe, and quantify. In particular, we find from our simulation results that the number of penguins in the huddle, the wind strength quantified by the Péclet number, and the amount of uncertainty in the penguin movement are the key factors governing the dynamics of a huddle. Our simulation results show that the features of this model are sufficient to result in each penguin having equal access to the benefits of the huddle.

To avoid prohibitively large computations and to allow for concise analysis, our model does not account for all possible details and scenarios. Rather, our goal is to provide a simple model, based on reasonable and well defined assumptions on the geometry of the huddle and the fluid mechanics of the wind. Our model recovers important features of actual huddles such as their overall shape, downwind motion, and an equal distribution of access to the benefits of the huddle among penguins. We describe the overall framework of our model in Method, including our assumptions. In Results and Discussion, we show simulation results, and identify, describe, and quantify the dynamics of the huddle. We discuss how these results compare to field observations and outline how one may extend this mathematical model to include a number of particular effects in Conclusion.

## Methods

In our mathematical model, individual heat preservation is the goal of each and every penguin in the huddle, irrespective of the heat preservation of the huddle as a whole. In other words, each penguin will move or stay stationary to minimize its own heat loss. This mathematical model therefore requires the determination of the local rate of heat loss for each penguin in the huddle. This local rate of heat loss depends on the temperature distribution outside the huddle which, in turn, depends on the wind flow around the huddle.

Specifically, we focus on the dynamics of a single huddle, where all the penguins present are part of the huddle. This huddle is assumed to be situated on a flat plane, so there are no obstacles impeding penguin movement. Penguins in this huddle have uniform size and shape. Our model does not account for all heat exchanges between penguins and their environments. In particular, penguins are known to lose a large quantity of heat through their feet and eyes [14]. In addition, the flow of wind over the top of the huddle contributes to cooling penguins. However, these heat loses affect penguins equally, irrespective of their position within a huddle, and therefore do not have a strong influence on the huddle dynamics. In contrast, the wind flow around the sides of the huddle affects penguins differently depending on whether they are on the edge or near the center of the huddle. For this reason, we model only the wind flowing on a two dimensional plane around the area occupied by the huddle. Our procedure to simulate huddles is as follows:

1. Generate a huddle and determine the huddle boundary.
2. Compute the wind flow around the huddle.
3. Compute the temperature profile around the huddle.
4. Compute the local rate of heat loss for each penguin.
5. Add random variations to the rate of heat loss (optional).
6. Identify the penguin with the highest rate of heat loss (the “mover”) and move it to a location on the boundary where heat loss is minimal.
7. Determine the new huddle boundary.
8. Repeat over the desired number of iterations by going back to Step 2.

In what follows, we describe in detail each step of this procedure. Included in this discussion are any simplifying assumptions, and their justification.

### 1. Generate a Huddle and Determine the Huddle Boundary

From observing videos of huddling penguins [13], [15], we note that huddling penguins are packed very tightly, presumably to minimize cold-body surfaces and maximize the ambient temperature in the huddle. It is well known mathematically that the densest packing of disks on a flat plane corresponds to having the center of each disk placed on a hexagonal or honeycomb lattice, so that each disk is inscribed in a regular hexagon and touches six neighboring disks. Moreover, direct observations reveal that huddling penguins generally position themselves on a hexagonal grid [13]. For these reasons, we assume in our model that penguins in the huddle stand centered on a hexagonal grid. Moreover, all points on the hexagonal lattice corresponding to the huddle’s interior are assumed to be occupied, so that there are no empty spaces within the huddle. Thus, the huddle boundary is determined uniquely by connecting the locations of penguins with fewer than six neighbors. Furthermore, we assume that all penguins in the huddle have at least two neighbors, in which case, the area generated by connecting the lattice points on the huddle boundary is a polygon. We therefore initiate our simulations by generating a huddle satisfying these conditions, starting with five penguins and adding penguins at locations chosen randomly among the eligible positions (adjacent to the huddle, with two neighbors, and leaving no empty space). Once the initial huddle is formed, we consider that the number of penguin within it remains constant.

### 2. Compute the Wind Flow Around the Huddle

To determine the wind flow around the huddle, we need only to consider a two dimensional flow around a polygon. Moreover, we assume that this flow is inviscid and irrotational. These assumptions imply that we do not resolve turbulent wind flows, which would obfuscate the computation of the temperature profile needed to compute the local rate of heat loss. Rather, we find a smooth, regular wind flow around the huddle. Nonetheless, the key relationships in this mathematical model between the wind, the temperature, and individual heat loss are not compromised by these assumptions.

Because the wind flow is significantly faster that the movement of a penguin, we assume that this flow is steady. In other words, the wind flow does not depend on the time elapsed since a penguin has relocated, and is only dependent on the huddle shape. Consequently, we are able to use the mathematics of complex variables and the physical theory of potential flow to describe the flow around the huddle.

Let 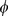 denote the wind velocity potential, and the wind velocity itself, 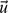, be the gradient of 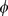: 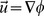. We combine the corresponding streamlines, denoted by 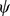, with 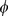 to form the complex potential, defined as 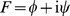 with i denoting the imaginary constant. Because the flow is irrotational, *F* is an analytic function on the flat plane outside the huddle. Since the huddle boundary is a polygon, we can make use of the Schwarz-Christoffel transformation, denoted by 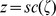. The Schwarz-Christoffel transformation is a conformal mapping from the outside of a disk, in what we will call the canonical domain denoted by 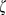, to the outside of a polygon, in what corresponds to the physical domain, denoted by 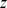 [16]. In the context of our model, the polygon in the physical domain corresponds to the boundary of the penguin huddle. This transformation is useful here because in the canonical domain, we are able to compute *F* easily via the Joukowsky transform 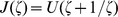, where *U* is the wind speed away from the disk. This transform maps the portion of the upper half-plane of the canonical domain outside the unit circle to the upper-half plane corresponding to 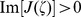 with 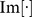 denoting the imaginary part. In this transformed domain, the complex potential is known exactly.

Mathematically, the wind flow around the polygon is therefore given by
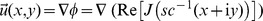
with 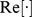 denoting the real part of a complex valued function and 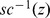 is the inverse of the Schwarz-Christoffel transformation. The key step in this computation is the Schwarz-Christoffel transformation. Because huddle shapes may be arbitrary polygons, we are not generally able to compute 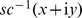 analytically. Instead, we use the freely available software package developed by Driscoll [17] which computes this transformation numerically. We then obtain a description of the wind flow at any point outside the huddle. Note that the wind speed, 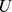, only affects the magnitude of the flow, but does not modify the location of the streamlines.

### 3. Compute the Temperature Profile Around the Huddle

The temperature far outside the huddle, denoted by 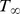, is significantly less than the temperature inside the huddle interior. The large transition in temperature from outside the huddle to inside the huddle is most significant near the huddle boundary where penguins are most exposed to the wind. To model this situation in our simulations, we assume that the temperature on the edge of the huddle, 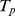, and 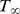 are constants with 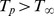. This assumption allows us to focus our attention solely on the temperature profile in the transition region near the huddle boundary.

Using the wind velocity around the huddle, 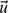, we solve for the temperature profile obtained from the advection-diffusion equation. As was the case for the wind flow, we assume that the temperature profile is steady, meaning that the temperature profile reaches equilibrium faster than any penguin motion. Consequently, the spatial distribution of the temperature around the huddle satisfies the steady advection-diffusion equation
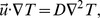
where 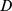 is the diffusivity of the temperature. This equation is invariant under conformal mapping provided 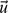 may be written as the gradient of a potential function. We can therefore use the Schwarz-Christoffel mapping once again and solve for the temperature around a disk in the canonical domain, before mapping it back around the polygonal huddle boundary. To simplify the analysis, we non-dimensionalize the equations by rescaling the velocity by the wind velocity away from the huddle 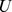, as 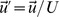. We rescale the temperature by the temperature difference between the penguins and the air away form the huddle 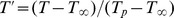, so that 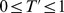. We also rescale distances by the size of the huddle 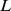, which corresponds to the radius of a huddle with the same number of penguins disposed to fill a disk, as 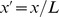, 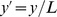. Rewriting the equation above in terms of these new variables, we obtain the non-dimensional equation



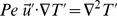
with 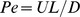 denoting the Péclet number. This non-dimensional number is proportional to the wind speed 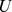 and therefore captures the effects of varying the strength of the wind. The boundary conditions we use to solve this equation in the canonical domain are 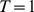 on the unit circle, where the penguins temperature remains effectively constant, and 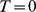 on a circle of radius 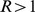, away from the huddle.

**Figure 1:**
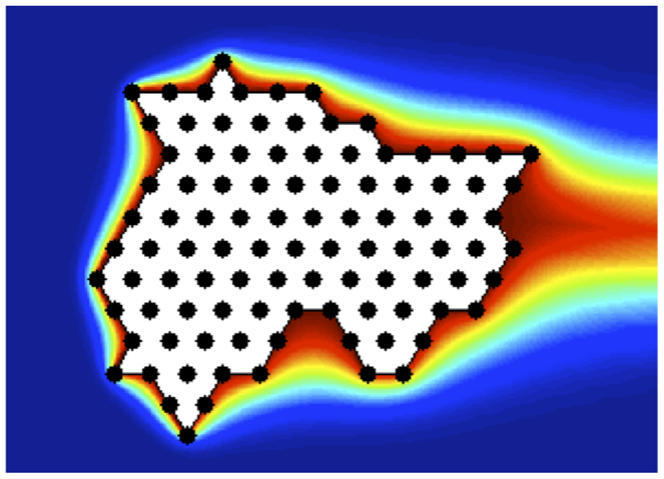
Sample of a computed temperature. Temperature distribution around a huddle of 100 penguins, for 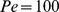. Here red and blue correspond to warmer and cooler temperatures, respectively. Individual penguins are shown in black, as is the boundary of the huddle, while the polygonal interior of the huddle is shown in white. We show an initial configuration where penguins have yet to be relocated.

We compute the temperature distribution exterior to the unit circle in the canonical domain by discretizing the annulus between the unit circle and the circle of radius 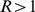 using finite differences. In particular, we discretize the annulus into regions of equal radius and angular increments. We then replace the differential operators in the equation with centered, finite difference approximations. The result leads to a linear system that we solve numerically. Using the Schwarz-Christoffel mapping, we may then recover the temperature distribution around the huddle. As an example, we show in Figure 1 a sample of the temperature profile obtained around a huddle made up of 100 penguins, choosing 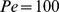.

When the flow around the huddle is turbulent, as is likely to be the case for all but the mildest winds [18], the relevant diffusivity 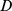 is a turbulent diffusivity. For that case, the relevant Péclet number is therefore a turbulent Péclet number. While this quantity varies depending on the flow, one may estimate it as being analogous to a turbulent Reynolds number, 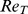, which is based on the diffusivity of momentum rather than temperature. Because the diffusion in turbulent flow is due to correlated fluid motions that mix temperature and momentum in similar manners, the turbulent Reynolds and Péclet numbers have similar values. The turbulent Reynolds number may be estimated as the value where the drag coefficient becomes effectively constant, which typically occurs for 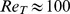 [19]. In the present study, we therefore limit our attention to flows with 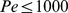.

### 4. Compute the Local Rate of Heat Loss for Each Penguin

Penguins at the huddle boundary experience the most significant heat loss compared to those within the huddle interior since the temperature profile changes most abruptly outside the huddle boundary. We seek to find the penguin on the huddle’s edge with the largest rate of heat loss. Therefore, we compute the local rate of heat loss only for penguins on the huddle boundary.

The local rate of heat loss at a boundary is proportional to the derivative of the temperature in the direction normal to the boundary [20]. From our computed temperature profile, we may approximate the normal derivative along the unit circle (parameterized by angle 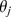) in the canonical domain using
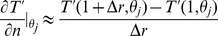
where 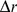 is the distance between consecutive points in the radial direction. To determine the heat loss associated to each penguin on the boundary, we first define points 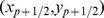 that are located halfway between consecutive penguins on the boundary, respectively labeled as penguins 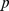 and 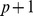. We then find the preimage of these points along the unit circle as 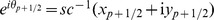. The heat loss associated to penguin 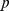 is then given by



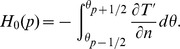
We approximate 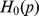 using the numerically obtained temperature profile obtained as described above to approximate the normal derivative, which we then integrate numerically. We note that 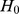 is always positive, indicating that heat is always lost. Penguins inside the huddle are assumed to lose only a negligible amount of heat compared to those on the periphery of the huddle, and we therefore set 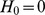 for penguins that are not on the edge of the huddle.

### 5. Add Random Variations (Optional)

The heat loss computation described above is idealized because everything is assumed to be known with absolute certainty. To account for variations that can occur in real huddles, we add uncertainty to this model through a random perturbation to the heat loss associated to each penguin on the boundary. We let
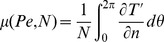
denote the average heat loss experienced by penguins disposed in a disk subject to the same conditions as the model huddle, with 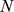 the number of penguins in the huddle. We introduce uncertainty in our mathematical model by assigning to each penguin an effective heat loss that includes a random component



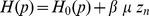
where 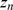 is a random number drawn from a uniform distribution ranging from −0.5 to 0.5, and 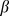 is a parameter quantifying the magnitude of the random component relative to that of the heat lost to the ambient air. While 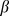 remains constant throughout the iterative process, 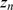 is re-drawn each time a penguin moves and the heat loss is recomputed. We then use the effective heat loss, 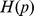, to determine the dynamics of the huddle.

The parameter 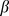 determines the importance of random variations in the system. The special case 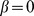 yields a completely deterministic, and idealized, system. No individual variations are captured in this case, but the results are perfectly replicable, which simplifies the analysis. As 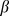 increases, the effects of random variations increase, and the importance of wind-related heat loss progressively diminishes. For large values of 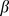, say for 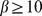, the wind direction would become negligible and model penguins would perceive a heat loss that is essentially random. It is likely that the relevant magnitude of 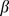 depends on the harshness of the conditions to which the huddle is subjected. In low winds or relatively warm temperature, the heat lost by each penguin is not very large, and penguin behavior is therefore likely to contain more random variations. On the other hand, in very harsh conditions, the imperative to minimize heat loss will be much stronger, and departure from the optimal behavior, in the form of random variations, are less likely.

### 6. Identify and Relocate the Mover

Among all penguins located at the huddle boundary, we call the penguin with the highest effective heat loss rate, corresponding to the largest value of 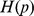, the “mover.” The mover vacates its current position and moves to a new position on the huddle boundary where the local heat loss rate is minimal. Because the huddle is situated on a flat plane, the mover can access any location around the huddle, but it cannot displace other penguins. This movement results in a generic motion from the windward side of the huddle to its leeward side.

In particular, the mover is relocated to a new position on the huddle boundary with at least two neighbors so that the huddle shape remains a polygon. The first neighbor is chosen as the penguin with the smallest heat loss rate corresponding to the smallest value of 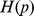. The second neighbor is chosen as the penguin experiencing the least heat loss among penguins adjacent to the first neighbor and on the huddle’s boundary.

Once the mover has been relocated, we may recompute the boundary of the huddle by connecting penguins with fewer than six neighbors. We then iterate the process outlined here by returning to step 2.

## Results and Discussion

### Deterministic Case (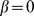)

**Figure 2:**
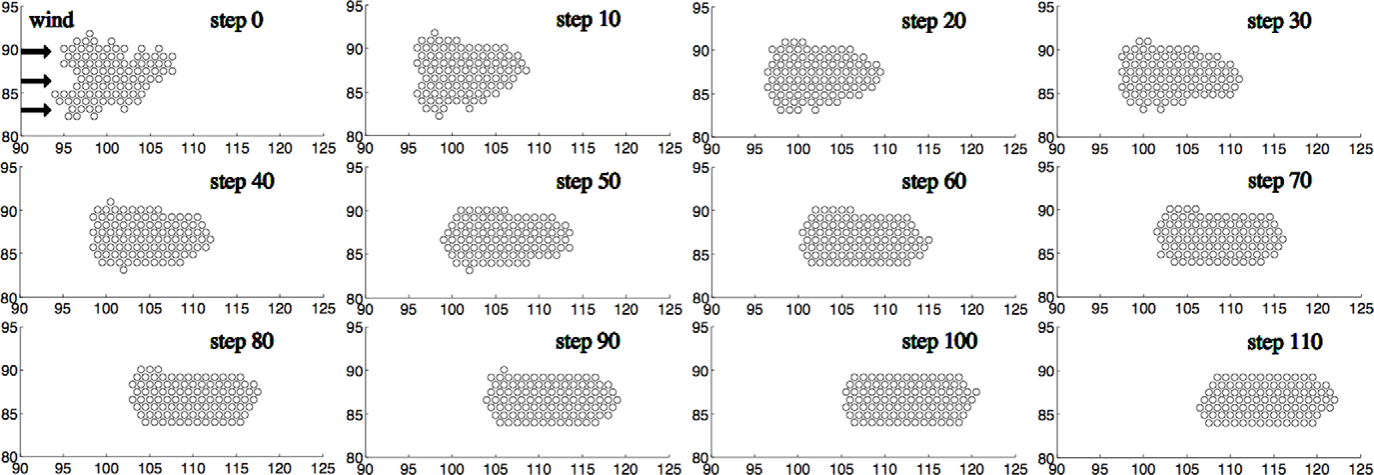
Time progression of a model huddle. Dynamics of a huddle according to our deterministic model, with parameters *N* = 100 penguins, *Pe* = 10, *R* = 4. The wind is coming from the left side.

We begin by looking at the progression of a single huddle in the absence of random perturbations, 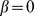. Figure 2 shows a huddle consisting of 100 penguins with 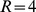 and a Péclet number of 10. Here the wind blows from left to right. One time step, or iteration, corresponds to the relocation of a single penguin from where heat loss is greatest to where it is minimized. Clearly the initial shape does little to conserve heat as too many individuals are exposed to the wind.

After only 10 steps, a more streamlined huddle starts to form as penguins on the windward side begin to relocate to the leeward side. Notice that the penguins located in thinner structures are the first to relocate, causing the initial indentations of the huddle to smooth out. As penguins continues to relocate, the huddle takes a somewhat elliptic shape. By the 50th time step, the outline of the final huddle formation is visible. During this phase, only penguins on the windward, upper, and lower edges of the huddle relocate. After 100 time steps, the huddle has reached its final shape, with flat sides and rounded windward and leeward ends. From here on, the thickness of the huddle remains constant. The huddle thus retains its basic structure while slowly traveling downwind. Moreover, regardless of their original position, every penguin eventually spends some time exposed to the wind, as layers of penguins on the windward side peel away and expose previously sheltered penguins.

We analyzed how the dynamics of the huddle are influenced by variations in the radius of a circle in the canonical domain where the temperature is assumed to match the temperature at infinity, 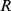, the number of penguins 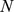, and the Péclet number, 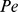, which characterizes the importance of inertial effects (wind) relative to that of diffusive effects (turbulent mixing). We begin by considering the effects of 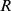, which has no physical significance (unless we consider an experiment where penguins huddle in a finite wind-tunnel). We studied the dynamics of huddles using values of 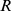 ranging from 1.5 to 30. For small values of 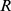, the temperature distribution was nearly uniform around the huddle, resulting in nearly circular huddles. As 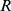 increased, heat loss became progressively greater in the windward direction. However, provided 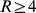, we found virtually no differences in the relative values of the normal derivative of the temperature along the huddle boundary. Because the dynamics of the huddle are only affected by the locations of maximal and minimal heat loss, the exact value of 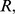 so long as it is greater than or equal to 4, has no impact on the dynamics of the huddle. This was confirmed by simulations of huddles of 100 penguins which converged to identical final configurations for various values of 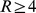. In the remainder of this study, we therefore fixed 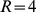.

**Figure 3:**
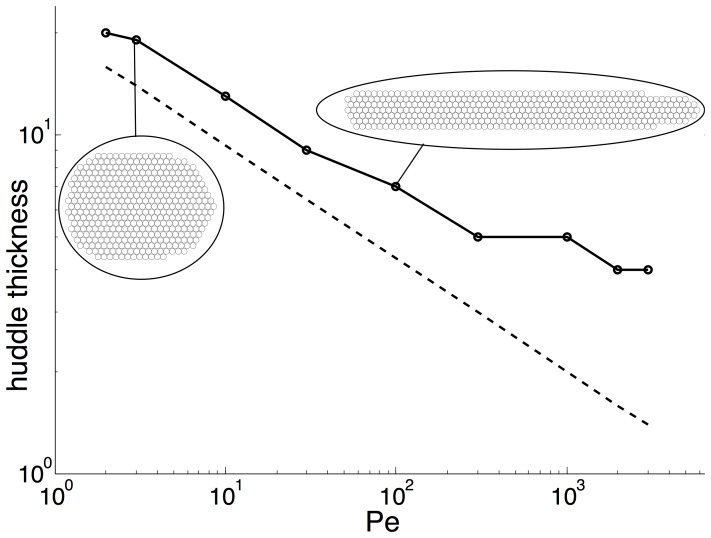
Dependence of the huddle shape on the Péclet number. Logarithm of the width, 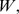 of model huddles consisting of *N* = 400 penguins as a function of the logarithm of the Péclet number (solid line) in the deterministic model. For comparison, the dashed line has equation 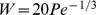.

The huddle dynamics show a stronger dependence on variations in the Péclet number. Figure 3 shows the width of a huddle consisting of 400 penguins for various Péclet numbers. For small Péclet numbers, when diffusive effects act to render the temperature distribution nearly uniform, the aspect ratio is close to one, depicting a more circular huddle. In contrast, for large Péclet numbers, where only a small leeward region is sheltered from the wind, the aspect ratio is much larger than one, depicting a more elongated huddle. As the Péclet number increases, the wind causes a stronger shift in the heat generated by the huddle. The wind pushes the heat distribution more narrowly around the huddle, causing the locations on the upper and lower edges of the huddle to exhibit more heat loss and thus be less attractive to relocating penguins. As a result, the huddle formation loses a layer of thickness and becomes more elongated.

To explain the observed dependence of the huddle thickness on 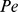, we consider the dependence of the thickness of the tail of warmer air that forms behind the huddle, 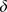, on the Péclet number. In this tail, the transport of heat by the wind scales, in dimensional form, as 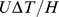, where 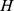 is the horizontal dimension of the huddle and 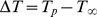. In steady-state, this transport is balanced by a diffusive transport, which scales as 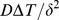. Equating those two expressions, we find that
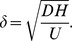
Since penguins in our model relocate based on minimizing their own heat loss, we estimate that the huddle thickness scales in the same manner as that of the warm downwind region. Applying this estimate and exploiting the conservation of area of the huddle, 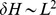, we conclude that the non-dimensional thickness of the huddle scales as



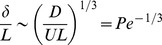
As shown in Figure 3, our results are consistent with this scaling, which appears as the dashed line, except in the regime where the huddle thickness is too small, at which point discrete effects are likely to obscure the dependence of the huddle shape on the Péclet number.

For a turbulent Péclet number of order 100, as can be expected in actual conditions, we find that the huddle formed by our model is fairly elongated, with a length-to-thickness ratio of approximately 8 (the huddle shown on the right of Figure 3 corresponds to 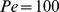). From observations of videos of huddling penguins [13], [15], we find that real huddles are more compact, indicating that the deterministic model does not exactly capture the actual huddling dynamics. Real huddles are also far less regular, indicating that random perturbations, which we discuss below, may play an important role.

**Figure 4:**
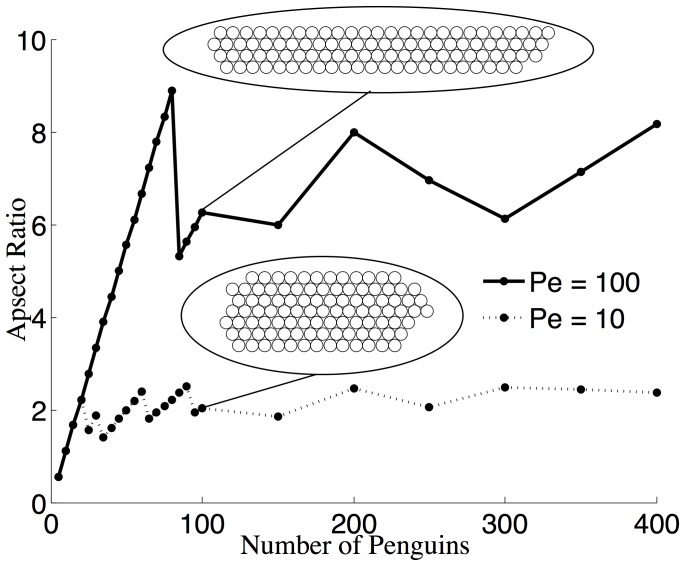
Dependence of the huddle shape on the number of penguins. Aspect ratios of huddles as a function of the number of penguins forming the huddle for fixed Péclet numbers, 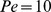 (dotted line) and 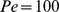 (solid line) in the deterministic model.

We also considered the effect of allowing the number of penguins, 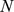, to vary (in the deterministic model). Figure 4 shows the aspect ratio of the huddle for a fixed Péclet number, either 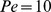 or 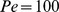. For small values of 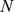, we see that increasing the number of penguins lengthens the huddle. Increasing the number of penguins increases the aspect ratio, as the length of the huddle grows while the width remains constant. Eventually, the huddle is sufficiently long for its sides to become well sheltered. Larger huddles are then thicker and the aspect ratio decreases. The tolerance for how elongated the huddle formation becomes before thickening depends on the Péclet number. For 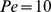, thicker huddles form, whereas for 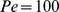, many more penguins must be present for the huddle to thicken, results that are consistent with those of Figure 4. We note that because the length and width of a huddle must increase by integer values, plots of the aspect ratio are relatively noisy. For small huddles, the change in the aspect ratio is more dramatic than for larger huddles. If the number of penguins exceeds 200, we see that the aspect ratio does not increase anymore and oscillates about a constant. Provided 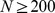 and for a fixed value of 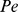, the overall shape of the huddle is then independent of 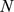, except for weak discrete size effects.

### Effects of Random Perturbations

**Figure 5:**
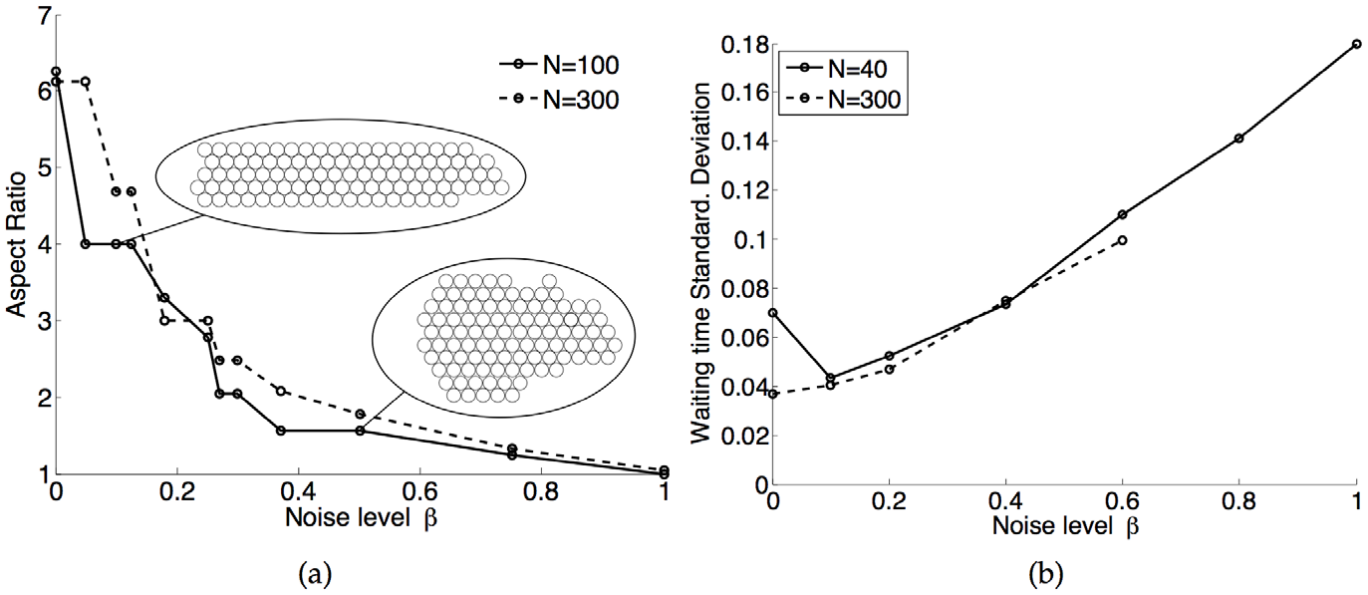
Influence of random variations on huddle shape and heat loss distribution. a) Aspect ratio of huddles of 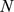 = 100 (solid line) and 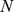 = 300 (dashed line) penguins for various values of the magnitude of the random perturbation added to the perceived heat loss, 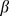. b) Dependence of the standard deviation of the normalized waiting time on 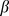. Other parameters are 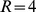 and 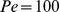 for both figures.

We investigated the effects of varying 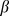, the magnitude of the random perturbation added to the perceived heat loss of each penguin. For 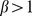, we observed very irregular huddles, with numerous protuberances, while values of 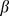 near 0 recovered results shown in the previous section. Figure 5a shows the aspect ratio of huddles of 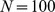 (solid line) and 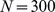 penguins (dashed line) as a function of the magnitude of the random perturbations. As 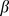 increases from 0, the huddle takes a shape similar to that observed in the absence of random variations, but with occasional random protuberances, which were excluded from the computation of the aspect ratio shown in Figure 5a. Large 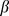, for which mobile penguins relocate randomly, result in irregular but essentially circular huddles, with an aspect ratio of one. Such huddles still tend to travel downwind, but much more slowly than in the deterministic case. Somewhat surprisingly, a value of 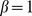 is sufficient to form nearly circular huddles. It is also noticeable that even relatively small perturbations, 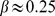, result in significantly thicker huddles. We found that huddles formed with 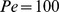 and 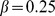 were comparable to those formed with 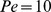 and 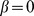. In addition, the early stages of noisy systems exhibited thicker huddles on the windward side than on the leeward side, indicating that the downwind side was more sensitive to random variations.

The deterministic system therefore appears to capture qualitative features of real penguin huddles, but it is also sensitive to random variations likely to occur through wind variations, uneven terrain, short-range motions within the huddle, and individual penguin perceptions. Incorporating even a modest level of random perturbations, 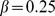, yields huddles whose shape matches qualitatively with observations. The irregularity of the huddle observed for large 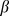 is consistent with observations of huddles in mild conditions, while smoother huddle, consistent with those observed in harsh conditions, are obtained for smaller values of 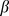. This behavior indicates that the importance of random variations is likely to decrease as conditions become harsher, down to a level near 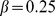.

### Equal Access to the Warmth of the Huddle

Finally, we investigated how evenly the burden of facing the wind was shared among penguins, and how sensitive the sharing of heat was to random perturbations. As a measure of how evenly heat losses were distributed, we consider the time interval required for each individual penguin to become the most exposed and therefore to relocate. In a huddle of 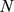 penguins, one would expect that an even distribution of heat would cause each penguin to relocate approximately every 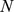 time steps. We recorded the standard deviation of the distribution of waiting times normalized by the mean waiting time of 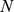. For comparison, if all penguins had an equal probability to relocate at every time step, we should find a mean normalized waiting time of 1, with a standard deviation of 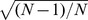, as the waiting time would then have a geometric distribution.

Figure 5b shows the standard deviation of the waiting time, 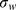, as a function of 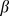 for huddles of 40 (solid line) and 300 (dashed line) penguins. For smaller huddles, in the absence of random variations, the huddle may enter a cycle which slightly increases variations in the waiting time. The addition of even a small level of random variations removes such exact cycles and quickly reduces the standard deviation of the waiting time. Larger huddles were less likely to enter into cyclic motion resulting in higher waiting time variations, and therefore 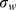 is seen to slowly increase monotonically with 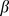 for 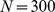. Large values of 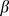 and 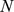 were difficult to simulate in our model, as empty spaces may form in the huddle when randomly relocating penguins enclose an open space. Such empty spaces do not appear for low values of 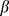, as relocating penguins then move to form as tight a huddle as possible. We note that the typical standard deviation of the waiting time for low levels of random variations, 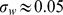, is far lower than what one would observe if the moving penguin was chosen perfectly randomly 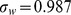 for 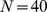 and 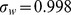 for 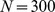. Even as 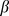 approaches 1, where nearly circular huddles formed, distribution of heat remains nearly even. This indicates that the heat loss experienced by all penguins in our model is very narrowly distributed, and thus very nearly as even as possible, for a wide range of random perturbations.

### Conclusion

By design, the model presented here contains only a minimal number of assumptions. Our model penguins are subject to heat loss in windy conditions, and they are simply trying to remain as warm as possible without resorting to displacing any of their fellow penguins. Our results were found to be robust to variations in initial conditions, and to the choice of the location where the temperature is assumed to match the temperature at infinity (i.e. the variable 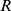).

By incorporating general observations, such as the hexagonal packing of huddles, and the usually long-range displacements of relocating penguins, our model reproduces several key features of the dynamics of huddles. By including a moderate random variation level of 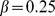, and using a turbulent Péclet number of approximately 100, the general shape of our huddles was found to be qualitatively similar to that of real huddles, as seen in Figure 5a. Huddles in our model also travel downwind, and their dynamics result in a relatively equal sharing of the heat loss among penguins. In addition, the equal heat sharing was found to be robust to variations in initial conditions and to random perturbations. The addition of a certain level of random variations was found to be necessary to obtain realistic looking huddles, both in their overall aspect ratio, and in their somewhat irregular boundary.

The even sharing of heat loss is of particular interest, as we find that even by modeling penguins that are only intent on minimizing their own exposure to the elements, a nearly uniform heat loss distribution can be achieved for the entire huddle. Our model does not rule out that individual penguins may at times sacrifice for the benefit of the huddle as a whole. Rather it emphasizes that such behavior is not essential to achieving an even distribution of the heat loss among penguins.

It is worth noting that the huddles obtained via our model for realistic values of 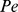 did not correspond to huddles shapes that would minimize heat loss for the huddle *as a whole*. In the absence of random variations, the huddles were too elongated, see Fig. 3, while random variations made the huddle boundary more irregular than is optimal, see Fig. 5a. This observation may be due to the limitations of the model, outlined in the model description, including the approximations made in estimating the heat losses. It may also indicate that penguins do not chose their location solely based on minimizing their own heat loss, but somehow take into account the benefit of surrounding penguins. It is also possible that even elongated or irregular huddles provide sufficient protection from the elements as to allow the survival of emperor penguins, or that penguins are simply unable to optimize heat loss for a huddle including hundreds of individuals. The advantage of our model is its ability to outline clear causal relations between our assumption that each penguins aims to minimize its own heat loss and the resulting huddle dynamics. However, this model by no means captures all the components of the behavior of huddling penguins.

There are several possible extensions that could make our model more realistic and that require further investigation. Firstly, the wind was assumed to remain constant, which is an idealization that could be relaxed by allowing the wind direction to vary over time. In addition, the wind velocity, captured by 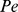, could also be made to be time dependent.

Secondly, the manner in which penguins relocate could be made more realistic by considering that penguins move downwind along the huddle boundary until they find a position deemed sufficiently sheltered, rather than find the absolute best available location. This would require the addition of a new parameter, a shelter threshold, which would determine at what point penguins stop looking for a better location. Another aspect that could be incorporated in future models is the presence of natural obstacles, such as cracks or icebergs, which could be simulated by preventing relocating penguins from moving to certain fixed locations. Tthe criterion used to determine which penguin has to relocate is currently based on instantaneous exposure rather than cumulative exposure. Using cumulative heat loss to determine which penguin relocates may be more appropriate than using instantaneous heat loss, as it would take into account the duration of the exposure. At present, our model computes only relative exposure, making an individual cumulative heat budget impractical. However, if one were willing to solve the governing equations in a manner that allows for the cumulative heat loss to be tracked, which would require significantly more computational effort, then the penguin that has lost the most heat cumulatively could be chosen as the “mover”.

Finally, it is possible to model a huddle in a continuous rather than discrete fashion, with its boundary 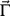 being a curve that moves over time. Its normal velocity could be proportional the local heat loss, to which a number 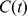 is added to preserve the total area of the huddle:
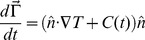
with 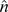 the local normal to the interface. Such an approach may be more amenable to analysis, and emphasizes the similarity between the dynamics on the windward side and those of melting [21], though the comparison does not extend to the leeward side, which behaves in the opposite way to that of freezing material. However, this approach does not track individual penguins, and may not be used to determine to what extent heat losses are evenly distributed.
